# Formalizing the LLL Basis Reduction Algorithm and the LLL Factorization Algorithm in Isabelle/HOL

**DOI:** 10.1007/s10817-020-09552-1

**Published:** 2020-06-09

**Authors:** René Thiemann, Ralph Bottesch, Jose Divasón, Max W. Haslbeck, Sebastiaan J. C. Joosten, Akihisa Yamada

**Affiliations:** 1 University of Innsbruck, Innsbruck, Austria; 2 University of La Rioja, Logroño, Spain

**Keywords:** Certified algorithm, Complexity verification, Lattices, Polynomial factorization, Shortest vector problem, Verified LLL implementation

## Abstract

The LLL basis reduction algorithm was the first polynomial-time algorithm to compute a reduced basis of a given lattice, and hence also a short vector in the lattice. It approximates an NP-hard problem where the approximation quality solely depends on the dimension of the lattice, but not the lattice itself. The algorithm has applications in number theory, computer algebra and cryptography. In this paper, we provide an implementation of the LLL algorithm. Both its soundness and its polynomial running-time have been verified using Isabelle/HOL. Our implementation is nearly as fast as an implementation in a commercial computer algebra system, and its efficiency can be further increased by connecting it with fast untrusted lattice reduction algorithms and certifying their output. We additionally integrate one application of LLL, namely a verified factorization algorithm for univariate integer polynomials which runs in polynomial time.

## Introduction

The LLL basis reduction algorithm by Lenstra, Lenstra and Lovász [17] is a remarkable algorithm with numerous applications. There even exists a 500-page book solely about the LLL algorithm [21], describing applications in number theory and cryptography, as well as the best known deterministic algorithm for factoring polynomials, which is used in many of today’s computer algebra systems. One immediate application of the LLL algorithm is to compute an approximate solution to the following problem:

Shortest Vector Problem (SVP): Given a linearly independent set of *m* vectors, $f_0,\ldots , f_{m-1}\in \mathbb {Z}^n$, which form a basis of the corresponding *lattice* (the set of vectors that can be written as linear combinations of the $f_i$, with integer coefficients), compute a non-zero lattice vector that has the smallest-possible norm.

A quick example showing that the problem can have quite non-trivial solutions is as follows (we will return to this example later).

### Example 1

Consider $f_0 = (1,1\,894\,885\,908,0)$, $f_1 = (0,1,1\,894\,885\,908)$, and $f_2 = (0,0,2\,147\,483\,648)$. The lattice of $f_0,f_1,f_2$ has a shortest vector $(-3,17,4)$, which can be written as the linear combination $-3f_0 + 5\,684\,657\,741f_1 - 5\,015\,999\,938f_2$.

In fact, finding an exact solution of SVP is NP-hard in general [20]. Nevertheless, the LLL algorithm takes any basis of a lattice *L* as input and outputs in polynomial-time a basis of *L* which is *reduced w.r.t.*$\alpha$, which implies, that the first of the output vectors is at most $\alpha ^{\frac{m-1}{2}}$ times larger than a shortest non-zero vector in the lattice. The parameter $\alpha >\frac{4}{3}$ also determines the running time.

In this paper we provide the first mechanized soundness proof of the LLL algorithm: the functional correctness is formulated as a theorem in the proof assistant Isabelle/HOL [24]. Since Isabelle code can be exported to other programming languages and then run on actual data, our work results in a verified implementation of the LLL algorithm. Having verified implementations of algorithms is important not mainly because the correctness of the algorithms themselves might be in doubt, but because such implementations can be composed into large reliable programs, of which every part has been formally proved to work as intended.

The proof of soundness consists of two phases: First, we prove that an abstract version of the algorithm, one that is inefficient in practice, is sound. Next, we refine the abstract version to obtain an optimized algorithm, and show that the output of the two versions coincides. Thus, we rely on the more easily provable soundness of the inefficient implementation, to derive the soundness of the optimized one.

We additionally provide a formal proof of a polynomial bound on the running-time of the algorithm: we first show a polynomial bound on the number of arithmetic operations, and then prove that the bit-representations of all numbers during the execution are polynomial in the size of the input.

We also include a formalization of an alternative approach to the reliable computation of reduced bases: getting a reduced basis using a fast external (unverified) tool, and then certifying the result using a verified checker. This approach, which we call the *certified* approach, runs 10$\times$ faster than the fully verified algorithm, and is even faster than Mathematica.

In addition to the LLL algorithm, we also verify one application, namely a polynomial-time algorithm for factoring univariate integer polynomials, that is: factorization into the content and a product of irreducible integer polynomials. It reuses most parts of the formalization of the Berlekamp–Zassenhaus factorization algorithm [7], where the main difference is that the exponential-time algorithm in the reconstruction phase is replaced by a polynomial-time procedure based on the LLL algorithm.

The whole formalization is based mainly on definitions and proofs from two books on computer algebra: [29, Chapter 16] and [21]. Thanks to this formalization effort, we were able to find a serious (but fixable) flaw in the factorization algorithm for polynomials as it is presented in [29].

Our formalization is available in the archive of formal proofs (AFP) [2, 9]. All definitions and lemmas found in this paper are also links which lead to an HTML version of the corresponding Isabelle theory file.

**Related work.** This work combines two conference papers [3, 8] in a revised and consistent manner. We have expanded the factorization part by providing more details about the bug detected in [29], the required modifications to fix it, as well as some optimizations. Moreover, the formalization of the certified approach is new, and new experiments comparing the performance of the verified algorithm, Mathematica, the certified approach, and a dedicated floating-point implementation are also provided.

We briefly discuss how the present work ties in with other related projects. As examples of verified software we mention a solver for linear recurrences by Eberl [10] and  [6, 26], a tool for checking untrusted termination proofs and complexity proofs. Both tools require computations with algebraic numbers. Although verified implementations of algebraic numbers are already available both in Coq [4] and in Isabelle/HOL [16, 18], there is still room for improvement: since the algebraic number computations heavily rely upon polynomial factorization, the verification of a fast factorization algorithm would greatly improve the performance of these implementations. A natural choice would then be van Hoeij’s algorithm [28], which is currently the fastest deterministic polynomial factorization algorithm. Since this algorithm uses LLL basis reduction as a subroutine, a future verified version of it can make full use of our verified, efficient LLL implementation.

**Structure of the work.** The remaining sections are organized as follows: Sect. 2 contains the preliminaries. We present the main ideas and algorithms for Gram–Schmidt orthogonalization, short vectors and LLL basis reduction in Sect. 3. The formalization and verification of these algorithms is discussed in Sect. 4. In Sect. 5 we discuss the details of an efficient implementation of the algorithms based on integer arithmetic. In Sect. 6 we illustrate the formal proof of the polynomial-time complexity of our implementation of the LLL algorithm. Section 7 explains how to invoke external lattice reduction algorithms and certify their result. In Sect. 8 we present experimental results, relating various verified and unverified implementations of lattice reduction algorithms. We present our verified polynomial-time algorithm for factoring integer polynomials in Sect. 9, and describe the flaw in the textbook. Finally, we conclude in Sect. 10.

## Preliminaries

We assume some basic knowledge of linear algebra, but recall some notions and notations. The inner product of two real vectors $v=(c_0,\dots ,c_n)$ and $w=(d_0,\dots ,d_n)$ is $v \bullet w = \sum _{i=0}^{n} c_i d_i$. Two real vectors are orthogonal if their inner product is zero. The Euclidean norm of a real vector *v* is $|\!|v|\!| = \sqrt{v\bullet v}$. A linear combination of vectors $v_0,\dots ,v_m$ is $\sum _{i=0}^{m} c_i v_i$ with $c_0,\dots ,c_m \in \mathbb {R}$, and we say it is an integer linear combination if $c_0,\dots ,c_m \in \mathbb {Z}$. A set of vectors is linearly independent if no element is a linear combination of the others. The span of a set of vectors is the vector space formed of all linear combinations of vectors from the set. If $\{v_0,\dots ,v_{m-1}\}$ is linearly independent, the spanned space has dimension *m* and $\{v_0,\dots ,v_{m-1}\}$ is a basis of it. The *lattice* generated by linearly independent vectors $v_0,\ldots ,v_{m-1} \in \mathbb {Z}^n$ is the set of linear combinations of $v_0,\ldots ,v_{m-1}$ with integer coefficients.

Throughout this paper we only consider univariate polynomials. The degree of a polynomial $f(x) = \sum _{i=0}^n c_i x^i$ with $c_n \ne 0$ is , the leading coefficient is , the content is the GCD of coefficients $\{c_0,\dots ,c_n\}$, and the norm $|\!|f|\!|$ is the norm of its corresponding coefficient vector, i.e., $|\!|(c_0,\dots ,c_n)|\!|$. A polynomial is primitive if its content is 1.

If $f = f_0 \cdot \ldots \cdot f_m$, then each $f_i$ is a factor of *f*, and is a proper factor if *f* is not a factor of $f_i$. Units are the factors of 1, i.e., $\pm 1$ in integer polynomials, and non-zero constants in field polynomials. By a factorization of a polynomial *f* we mean a decomposition $f = c \cdot f_0 \cdot \ldots \cdot f_m$ into the content *c* and irreducible factors $f_0,\dots ,f_m$; here irreducibility means that each $f_i$ is not a unit and admits only units as proper factors.

Our tool of choice for the formalization of proofs is Isabelle/HOL. Throughout the paper we simply write ‘Isabelle’ to refer to Isabelle/HOL. We assume familiarity with it, and refer the reader to [23] for a quick introduction. We also briefly review some Isabelle notation, in order to make most of the Isabelle code in the paper accessible to readers familiar only with standard mathematical notation.

All terms in Isabelle must have a well-defined type, specified with a double-colon: . Type variables have a  sign before the identifier. The type of a function with domain  and range  is specified as . Each of the base types , , and  corresponds to $\mathbb {N}$, $\mathbb {Z}$, and $\mathbb {Q}$, respectively. Access to an element of a vector, list, or array is denoted, respectively, by , , . For example, if  is of type , the type of lists of vectors of integers, then  denotes the *j*-th component of the *i*-th vector in the list. In the text, however, we will often use more convenient mathematical notations instead of Isabelle’s notations. For example, we write $f_i$ rather than . The syntax for function application in Isabelle is ; terms are separated by white spaces, and  can be either the name of a function or a lambda expression. Some terms that we index with subscripts in the in-text mathematical notation are defined as functions in the Isabelle code (for example $\mu _{i,j}$ stands for ). Isabelle keywords are written in  font, and comments are embraced in .

At some points, we use locales [1] to ease the development of the formal proofs. Locales are detached proof contexts with fixed sets of parameters and assumptions, which can be later reused in other contexts by means of so-called interpretations. The  keyword is used to set a block of commands, delimited by  and , as part of an existing locale. It can also be used to declare anonymous proof contexts. Locales can be seen as permanent contexts.

## The LLL Basis Reduction Algorithm

In this section we give a brief overview of the LLL Basis Reduction Algorithm and the formalization of some of its main components in Isabelle/HOL.

### Gram–Schmidt Orthogonalization and Short Vectors

The Gram–Schmidt orthogonalization (GSO) procedure takes a list of linearly independent vectors $f_0,\ldots ,f_{m-1}$ from $\mathbb {R}^n$ or $\mathbb {Q}^n$ as input, and returns an orthogonal basis $g_0,\ldots ,g_{m-1}$ for the space that is spanned by the input vectors. The vectors $g_0,\ldots ,g_{m-1}$ are then referred to as *GSO vectors*. To be more precise, the GSO is defined by mutual recursion as:

1 $$\begin{aligned} g_i&{:=} f_i - \sum _{j< i} \mu _{i,j}g_j&\mu _{i,j}&{:=} {\left\{ \begin{array}{ll} 1 &{} \text {if }i = j \\ 0 &{} \text {if }j > i \\ \frac{f_i \bullet g_j}{|\!|g_j|\!|^2} &{} \text {if }j < i \end{array}\right. } \end{aligned}$$

An intuition for these definitions is that if we remove from some $f_i$ the part that is contained in the subspace spanned by $\{f_0,\ldots ,f_{i-1}\}$, then what remains (the vector $g_i$) must be orthogonal to that subspace. The $\mu _{i,j}$ are the coordinates of $f_i$ w.r.t. the basis $\{g_0,\ldots , g_{m-1}\}$ (thus $\mu _{i,j}=0$ for $j>i$, since then $g_j\perp f_i$).

The GSO vectors have an important property that is relevant for the LLL algorithm, namely they are short in the following sense: for every non-zero integer vector *v* in the lattice generated by $f_0,\ldots ,f_{m-1}$, there is some $g_i$ such that $|\!|g_i|\!| \le |\!|v|\!|$. Moreover, $g_0 = f_0$ is an element in the lattice. Hence, if $g_0$ is a short vector in comparison to the other GSO vectors, then $g_0$ is a short lattice vector.

The importance of the above property of the Gram-Schmidt orthogonalization motivates the definition of a *reduced basis*, which requires that the GSO vectors be nearly sorted by their norm.

#### Definition 1

Let $\alpha \ge 1$. We say that a basis $f_0,\ldots ,f_{m-1}$ is *reduced w.r.t.*$\alpha$, if the GSO vectors satisfy $|\!|g_{i-1}|\!|^2 \le \alpha |\!|g_{i}|\!|^2$ for all $1< i < m$ and moreover $|\mu _{i,j}| \le \frac{1}{2}$ holds for all $j< i < m$.

The requirement on the $\mu _{i,j}$ implies that the *f*-vectors are nearly orthogonal. (If $|\mu _{i,j}| = 0$ for all $j< i < m$, then the *f*-vectors are pairwise orthogonal.)

The connection between a reduced basis and short vectors can now be seen easily: If $f_0,\ldots ,f_{m-1}$ is reduced, then for any non-zero lattice vector *v* we have

2 $$\begin{aligned} |\!|f_0|\!|^2 = |\!|g_0|\!|^2 \le \alpha ^{m-1} \min \{ |\!|g_i|\!|^2 \mid 0 \le i < m\} \le \alpha ^{m-1} |\!|v|\!|^2, \end{aligned}$$

and thus, $|\!|f_0|\!| \le \alpha ^{\frac{m-1}{2}} |\!|v|\!|$ shows that $f_0$ is a short vector which is at most $\alpha ^{\frac{m-1}{2}}$ longer than the shortest vectors in the lattice.

#### Example 2

Consider the vectors $f_0,f_1,f_2$ of Example 1. The corresponding GSO vectors are

$$\begin{aligned} g_0&= (1, 1894885908, 0) \\ g_1&= \left( \frac{- 1894885908}{3590592604336984465}, \frac{1}{3590592604336984465}, 1894885908\right) \\ g_2&= \left( \frac{7710738904443408018070044672}{12892355250319448667906759645314351761},\right. \\&\quad \frac{-4069236502255632384}{12892355250319448667906759645314351761},\\&\quad \left. \frac{2147483648}{12892355250319448667906759645314351761}\right) . \end{aligned}$$

This basis is not reduced for any reasonable $\alpha$, since the norms $|\!|g_0|\!| \approx 2 \times 10^9 \approx |\!|g_1|\!|$ and $|\!|g_2|\!| \approx 6 \times 10^{-10}$ show that $g_2$ is far shorter than $g_0$ and $g_1$.

#### Example 3

Consider the vectors $f_0 = (-3, 17, 4)$, $f_1 = (-8480, -811, -2908)$ and $f_2 = (1290, 3351, -13268)$. The corresponding GSO vectors are

$$\begin{aligned} g_0&= (-3, 17, 4) \\ g_1&= \left( \frac{- 2662657}{314}, \frac{-255011}{314}, \frac{-456598}{157}\right) \\ g_2&= \left( \frac{99196564668416}{25441719249},\frac{91577292685312}{25441719249}, \frac{-314806070411264}{25441719249}\right) . \end{aligned}$$

This basis is reduced for every $\alpha \ge 1$, since the norms $|\!|g_0|\!| \approx 18$, $|\!|g_1|\!| \approx 9001$ and $|\!|g_2|\!| \approx 13463$ are sorted and $|\mu _{i,j}| \le \frac{1}{2}$ is satisfied for every $j< i < 3$.

In a previous formalization [27] of the Gram–Schmidt orthogonalization procedure, the $\mu$-values are computed implicitly. Since for the LLL algorithm we need the $\mu$-values explicitly, we implement a new version of GSO. Here the dimension *n* and the input basis  are fixed as locale parameters. The  are given here as rational vectors, but the implementation is parametric in the type of field. 
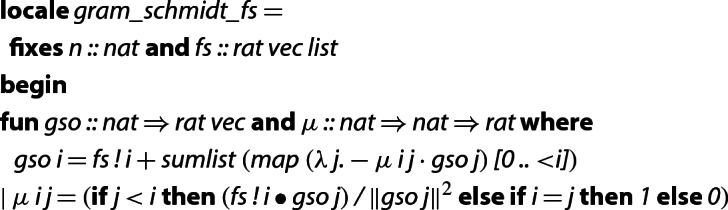
 It is easy to see that these Isabelle functions compute the *g*-vectors and $\mu$-values precisely according to their defining Eq. (1).

Based on this new formal definition of GSO with explicit $\mu$-values, it is now easy to formally define a reduced basis. Here, we define a more general notion, which only requires that the first *k* vectors form a reduced basis. 
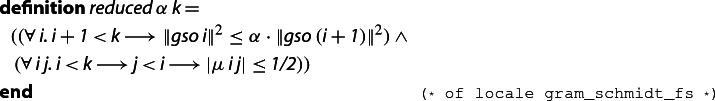


### LLL Basis Reduction

The LLL algorithm modifies the input $f_0,\dots ,f_{m-1} \in \mathbb {Z}^n$ until the corresponding GSO is reduced w.r.t. $\alpha$, while preserving the generated lattice. The *approximation factor*$\alpha$ can be chosen arbitrarily as long as $\alpha > \frac{4}{3}$.^1^

In this section, we present a simple implementation of the algorithm given as pseudo-code in Algorithm 1, which mainly corresponds to the LLL algorithm in a textbook [29, Chapters 16.2–16.3] (the textbook fixes $\alpha = 2$ and $m = n$). Here, $\lfloor x \rceil = \lfloor x + \frac{1}{2}\rfloor$ is the integer nearest to *x*. Note that whenever $\mu _{i,j}$ or $g_i$ are referred to in the pseudo-code, their values are computed (as described in the previous subsection) for the *current* values of $f_0,\ldots ,f_{m-1}$.
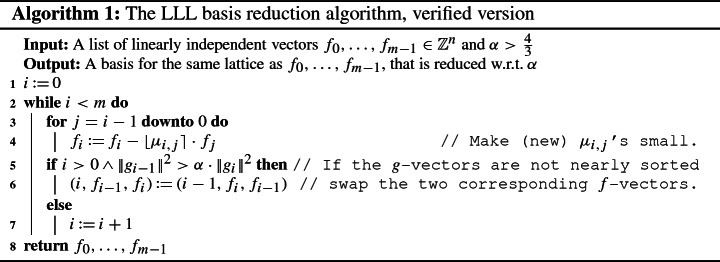


#### Example 4

On input $f_0, f_1, f_2$ from Example 1, with $\alpha = \frac{3}{2}$, the LLL algorithm computes the reduced basis given in Example 3.

We briefly explain the ideas underpinning Algorithm 1: Lines 3–4 work towards satisfying the second requirement for the basis to be reduced (see Definition 1), namely that the $\mu _{i,j}$ values be small. This is done by “shaving off”, from each $f_i$, the part that overlaps with some part of an $f_j$ (with $j<i$). This ensures that when the GSO is computed for this new basis, a (norm-wise) significant part of each $f_i$ does *not* lie in the subspace spanned by the $f_j$ with $j<i$ (as those parts have already been removed in line 4). When a violation of the first requirement for being a reduced basis is detected in line 5, the algorithm attempts to rectify this by performing a swap of the corresponding *f*-vectors in the next line. Thus, the algorithm continually attempts to fix the basis in such a way that it satisfies both requirements for being reduced, but the fact that it always succeeds, and in polynomial-time, is not obvious at all. For a more detailed explanation of the algorithm itself, we refer to the textbooks [21, 29].

In order to formalize the main parts of Algorithm 1, we first encode it in several functions, which we list and explain below. We are using a locale that fixes the approximation factor $\alpha$, the dimensions *n* and *m*, and the basis $\textit{fs}_{init}$ of the initial (input) lattice. 
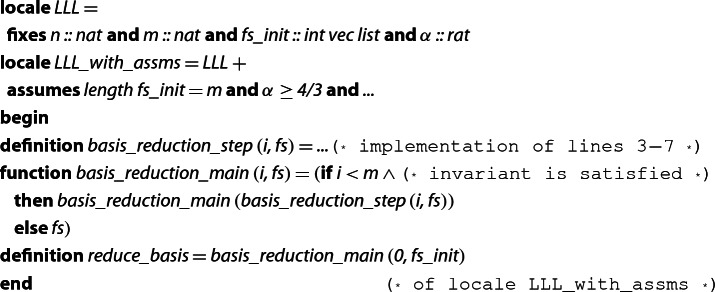


The following are some remarks regarding the above code fragments:

- The body of the while-loop (lines 3–7) is modeled by the function , whose details we omit here, but can be seen in the formalization.
- We remark that the actual Isabelle sources also contain an optimization that makes it possible to skip the execution of lines 3–4 in some cases when it can be determined that the $\mu$-values are already small. This optimization is explained in more detail in Sect. 5.
- The while-loop itself (line 2) is modeled as the function . The termination of the function will be proved later. Here, it is essential that invalid inputs do not cause nontermination: bad choices of $\alpha$ are prohibited by locale assumptions, and invalid inputs of  result in immediate termination by checking an invariant in every iteration of the loop.
- Finally, the full algorithm is implemented as the function , which starts the loop and then returns the final integer basis $f_0,\ldots ,f_{m-1}$.

In this section we only looked at how the algorithms were specified in Isabelle. In the next section we discuss the formal proofs of their soundness.

## Soundness of the LLL Basis Reduction Algorithm

### Gram–Schmidt Orthogonalization and Short Vectors

As mentioned in the previous section, the GSO procedure itself has already been formalized in Isabelle as a function called , in way of proving the existence of Jordan normal forms [27]. That formalization uses an explicit carrier set to enforce that all vectors are of the same dimension. For the current formalization task, the use of a carrier-based vector and matrix library is necessary: encoding dimensions via types [15] is not expressive enough for our application; for instance for a given square matrix of dimension *n* we need to multiply the determinants of all submatrices that only consider the first *i* rows and columns for all $1 \le i \le n$.

Below, we summarize the main result that is formally proved about  [27]. For the following code, we open a context assuming common conditions for invoking the Gram–Schmidt procedure, namely that  is a list of linearly independent vectors, and that  is the GSO of . Here, we also introduce our notion of linear independence for lists of vectors, based on an definition of linear independence for sets from an AFP-entry of H. Lee about vector spaces.
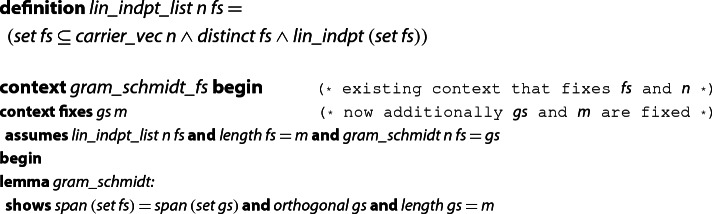


Unfortunately, lemma  does not suffice for verifying the LLL algorithm, since it works basically as a black box. By contrast, we need to know how the GSO vectors are computed, that the connection between  and  can be expressed via a product of matrices, and we need the recursive equations to compute  and the $\mu$-values. In order to reuse the existing results on , we first show that both definitions are equivalent. 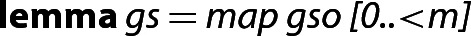


The connection between the *f*-vectors, *g*-vectors and the $\mu$-values is expressed by the matrix identity

3 $$\begin{aligned} \begin{bmatrix}f_0 \\ \vdots \\ f_{m-1}\end{bmatrix}&= \begin{bmatrix}\mu _{0,0} &{} \ldots &{} \mu _{0,m-1}\\ \vdots &{} \ddots &{} \vdots \\ \mu _{m-1,0} &{} \ldots &{} \mu _{m-1,m-1}\end{bmatrix} \cdot \begin{bmatrix}g_0 \\ \vdots \\ g_{m-1}\end{bmatrix} \end{aligned}$$

by interpreting the $f_i$’s and $g_i$’s as row vectors.

While there is no conceptual problem in proving the matrix identity (3), there are some conversions of types required. For instance, in lemma ,  is a list of vectors; in (1), *g* is a recursively defined function from natural numbers to vectors; and in (3), the list of $g_i$’s is seen as a matrix. Consequently, the formalized statement of (3) contains conversions such as  and , which convert a function and a list of vectors, respectively, into a matrix. In any case, the overhead is small and very workable; only a few lines of easy conversions are added when required. An alternative approach could be to do everything at the level of matrices [13]. 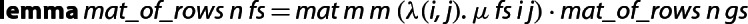


As mentioned in Sect. 3.1, our main use of GSO with regards to the LLL algorithm is that the norm of the shortest GSO vector is a lower bound on the norms of all lattice vectors. While proving this fact requires only a relatively short proof on paper, in the formalization we had to expand the condensed paper-proof into 170 lines of more detailed Isabelle source, plus several auxiliary lemmas. For instance, on paper one easily multiplies two sums ($(\sum \ldots ) \cdot \sum \ldots = \sum \ldots$) and directly omits quadratically many neutral elements by referring to orthogonality, whereas we first had to prove this auxiliary fact in 34 lines. 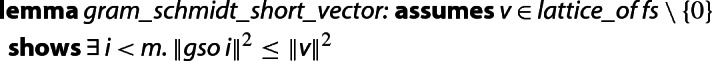


With the help of this result it is straight-forward to formalize the reasoning in (2) to obtain the result that the first vector of a reduced basis is a short vector in the lattice. 
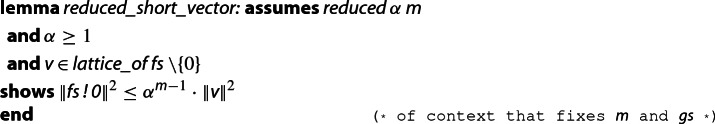


Finally, we mention the formalization of a key ingredient in reasoning about the LLL algorithm: orthogonal projections. We say $w \in \mathbb {R}^n$ is a projection of $v\in \mathbb {R}^n$ into the orthogonal complement of $S \subseteq \mathbb {R}^n$, or just *w* is an *oc-projection* of *v* and *S*, if $v - w$ is in the span of *S* and *w* is orthogonal to every element of *S*: 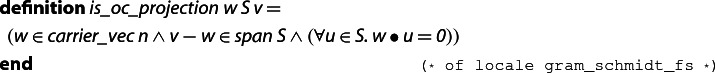


Returning to the GSO procedure, we prove that $g_j$ is the unique oc-projection of $f_j$ and $\{f_0,\dots ,f_{j-1}\}$. Hence, $g_j$ is uniquely determined in terms of $f_j$ and the span of $\{f_0,\dots ,f_{j-1}\}$. Put differently, we obtain the same $g_j$ even if we modify some of the first *j* input vectors of the GSO: only the span of these vectors must be preserved. This result is in particular important for proving that only $g_{i-1}$ and $g_i$ can change in Line 6 of Algorithm 1, since for any other $g_j$, neither $f_j$ nor the set $\{f_0,\dots ,f_{j-1}\}$ is changed by a swap of $f_{i-1}$ and $f_i$.

### LLL Basis Reduction

In this subsection we give an overview of the formal proof that Algorithm 1 terminates on valid inputs, with an output that has the desired properties.

In order to prove the correctness of the algorithm, we define an *invariant*, which is simply a set of conditions that the current state must satisfy throughout the entire execution of the algorithm. For example, we require that the lattice generated by the original input vectors $f_0,\ldots ,f_{m-1}$ be maintained throughout the execution of the algorithm. Intuitively this is obvious, since the basis is only changed in lines 4 and 6, and swapping two basis vectors or adding a multiple of one basis vector to another will not change the resulting lattice. Nevertheless, the formalization of these facts required 170 lines of Isabelle code.

In the following Isabelle statements we write  as a short form of , i.e., the Isabelle expression for the predicate  w.r.t. $\alpha$, considering the first  vectors, within the locale  with an . Similarly, we write  and  when we are referring to the $\mu$-values and the GSO vectors corresponding to the basis . 
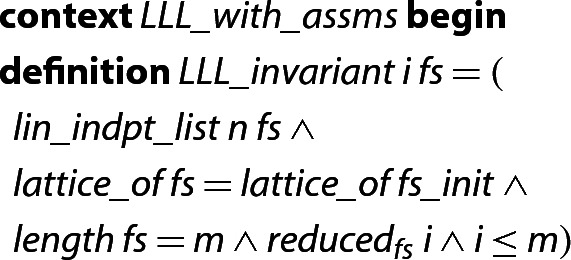
 The key correctness property of the LLL algorithm is then given by the following lemma, which states that the invariant is preserved in the while-loop of Algorithm 1 ( and  in the definition above refer to the variables with the same name in the main loop of the algorithm). Specifically, the lemma states that if the current state (the current pair ), prior to the execution of an instruction, satisfies the invariant, then so does the resulting state after the instruction. It also states a decrease in a measure, which will be defined below, to indicate how far the algorithm is from completing the computation—this is used to prove that the algorithm terminates. 
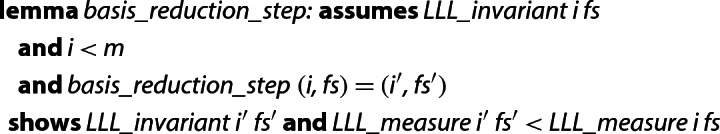


Using Lemma , one can prove the following crucial properties of the LLL algorithm.

1. The resulting basis is reduced and spans the same lattice as the initial basis. 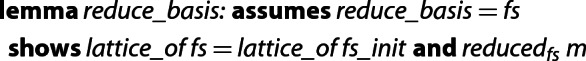
2. The algorithm terminates, since the  is decreasing in each iteration.
3. The number of loop iterations is bounded by  when invoking the algorithm on inputs  and . Therefore,  requires at most  many iterations.

Both the fact that the algorithm terminates and the fact that the invariant is maintained throughout its execution, are non-trivial to prove, as both proofs require equations that determine how the GSO will change through the modification of $f_0,\ldots ,f_{m-1}$ in lines 4 and 6. Specifically, we formally prove that the GSO remains unchanged in lines 3–4, that a swap of $f_{i-1}$ and $f_i$ will at most change $g_{i-1}$ and $g_i$, and we provide an explicit formula for calculating the new values of $g_{i-1}$ and $g_i$ after a swap. In these proofs we require the recursive definition of the GSO as well as the characterization via oc-projections.

In the remainder of this section, we provide details on the termination argument. The measure that is used for proving termination is defined below using *Gramian determinants*, a generalization of determinants which also works for non-square matrices. The definition of the measure is also the point where the condition $\alpha > \frac{4}{3}$ becomes important: it ensures that the base $\frac{4\alpha }{4 + \alpha }$ of the logarithm is strictly greater than 1.^2^
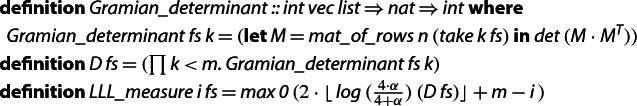


In the definition, the matrix  is the  submatrix of  corresponding to the first  elements of . Note that the measure is defined in terms of the variables  and . However, for lines 3–4 we only proved that  and the GSO remain unchanged. Hence the following lemma is important: it implies that the measure can also be defined purely from  and the GSO of , and that the measure will be positive. 
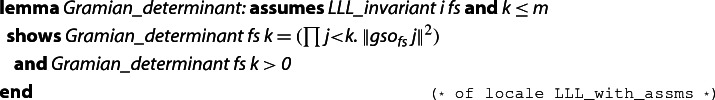


Having defined a suitable measure, we sketch the termination proof: The value of the Gramian determinant for parameter $k \ne i$ stays identical when swapping $f_i$ and $f_{i-1}$, since it just corresponds to an exchange of two rows, which will not modify the absolute value of the determinant. The Gramian determinant for parameter $k = i$ can be shown to decrease, by using the first statement of lemma , the explicit formula for the new value of $g_{i-1}$, the condition $|\!|g_{i-1}|\!|^2 > \alpha \cdot |\!|g_i|\!|^2$, and the fact that $|\mu _{i,i-1}| \le \frac{1}{2}$.

## An Efficient Implementation of the LLL Basis Reduction Algorithm

In the previous section we described the formalization of the LLL algorithm, which can already serve as a verified implementation of the algorithm. For the performance of the executable code obtained from the formalization, however, implementation-specific choices, such as how numbers should be represented, can have a huge impact. For example, working with rational numbers, represented as pairs of integers, incurs a huge performance penalty due to the need to perform a gcd computation after each operation, in order to reduce the resulting fraction and prevent a blow-up in the size of the denominators. To make this more concrete, one of our earlier implementations, based on rational number arithmetic, spent over 80% of the running time on gcd computations.

These considerations motivate us to opt for a version of the LLL algorithm that avoids the use of rationals, instead using only integers. One obstacle is that both the GSO vectors and the $\mu$-matrix usually consist of non-integral rational numbers. This is where Gramian determinants come into play once again.

For brevity of notation, we henceforth denote  by $d_k$ or , unless we wish to emphasize that $d_k$ is defined as a determinant. Here, for  we often omit the implicit parameter  if it is clear from the context. We also adopt the convention that $d_0 = 1$.

The most important fact for the integer implementation is given by the following lemma. It states that although the $\mu$-values themselves will not be integers in general, multiplying each of them by an appropriate Gramian determinant will always result in an integer. 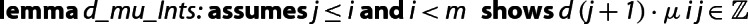


Based on this fact we derive a LLL implementation which only tracks the values of $\tilde{\mu }$, where $\tilde{\mu }_{i,j} {:=} d_{j+1}\mu _{i,j}$ (in the Isabelle source code, $\tilde{\mu }$ is called ). We formally prove that the $\tilde{\mu }$ values can be calculated using only integer arithmetic, and that it suffices to keep track of only these values in the LLL algorithm.

### Gram–Schmidt Orthogonalization

In order to obtain a full integer-only implementation of the LLL algorithm, we also require such an implementation of the Gram–Schmidt orthogonalization. For this, we mainly follow [12], where a GSO-algorithm using only operations in an abstract integral domain is given. We implemented this algorithm for the integers and proved the main soundness results following [12].
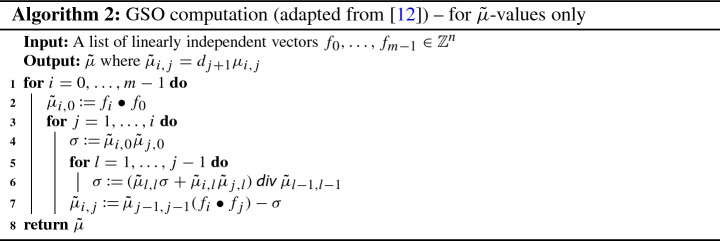


The correctness of Algorithm 2 hinges on two properties: that the calculated $\tilde{\mu }_{i,j}$ are equal to $d_{j + 1}\mu _{i,j}$, and that it is sound to use integer division  in line 6 of the algorithm (in other words, that the intermediate values computed at every step of the algorithm are integers). We prove these two statements in Isabelle by starting out with a more abstract version of the algorithm, which we then refine to the one above. Specifically, we first define the relevant quantities as follows: 
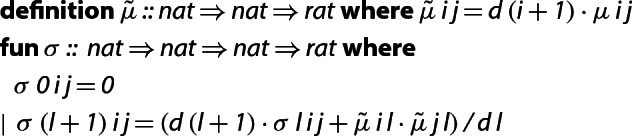
 Here $\tilde{\mu }$ is not computed recursively, and  represents the value of $\sigma$ at the beginning of the *l*-th iteration of the innermost loop, i.e.,  is the value of $\sigma$ after executing line 4. We remark that the type of (the range of) $\tilde{\mu }$ and of $\sigma$ is , rather than ; this is why we can use general division for fields (/) in the above function definition, rather than integer division (). The advantage of letting  and  return rational numbers is that we can proceed to prove all of the equations and lemmas from [12] while focusing only on the underlying mathematics, without having to worry about non-exact division. For example, from the definition above we can easily show the following characterization. 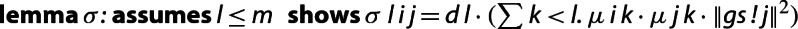 This lemma is needed to prove one of the two statements that are crucial for the correctness of the algorithm, namely that the computation of $\tilde{\mu }$ in lines 2 and 7 is correct (recall the identities $d_0 = 1$ and $d_j = \tilde{\mu }_{j-1,j-1}$ for $j > 0$). 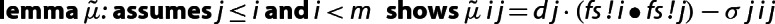


To prove that the above quantities are integers, we first show $d_i g_{i}\in {\mathbb {Z}}^n$. For this, we prove that $g_i$ can be written as a sum involving only the *f* vectors, namely, that $g_i = f_i - \sum _{j< i} \mu _{i,j}g_j = f_i - \sum _{j < i} \kappa _{i,j} f_j$. Two sets of vectors $f_0,\ldots ,f_{i-1}$ and $g_0,\ldots ,g_{i-1}$ span by construction the same space and both are linearly independent. The $\kappa _{i,j}$ are therefore simply the coordinates of $\sum _{j < i} \mu _{i,j}g_j$ in the basis $f_0,\ldots ,f_{i-1}$. Now, since the *f* vectors are integer-valued, it suffices to show that $d_i \kappa _{i,j} \in {\mathbb {Z}}$, in order to get $d_i g_{i} \in {\mathbb {Z}}^n$. To prove the former, observe that each $g_i$ is orthogonal to every $f_l$ with $l < i$ and therefore $0 = f_l \bullet g_i = f_l \bullet f_i - \sum _{j < i} \kappa _{i,j}(f_l \bullet f_j)$. Thus, the $\kappa _{i,j}$ form a solution to a system of linear equations:The coefficient matrix *A* on the left-hand side where $A_{i,j} = f_i \bullet f_j$ is exactly the Gramian matrix of  and . By an application of Cramer’s lemma,^3^ we deduce:The matrix , which is obtained from  by replacing its -th column by , contains only inner products of the *f* vectors as entries and these are integers. Then the determinant is also an integer and $d_i \kappa _{i,j} \in \mathbb {Z}$.

Since $\mu _{i,j}=\frac{f_i \bullet g_j}{|\!|g_j|\!|^2}$ and $\frac{d_{j+1}}{d_j} = |\!|g_j|\!|^2$, the theorem  from the introduction of this section, stating that $\tilde{\mu }_{i,j} = d_{j+1} \mu _{i,j}\in {\mathbb {Z}}$, is easily deduced from the fact that $d_i g_{i} \in {\mathbb {Z}}^n$.

In our formalization we generalized the above proof so that we are also able to show that $d_l(f_i - \sum _{j<l}\mu _{i,j} g_j)$ is integer-valued (note that the sum only goes up to *l*, not *i*). This generalization is necessary to prove that all $\sigma$ values are integers. 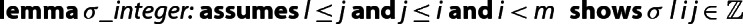


Having proved the desired properties of the abstract version of Algorithm 2, we make the connection with an actual implementation on integers that computes the values of $\tilde{\mu }$ recursively using integer division.
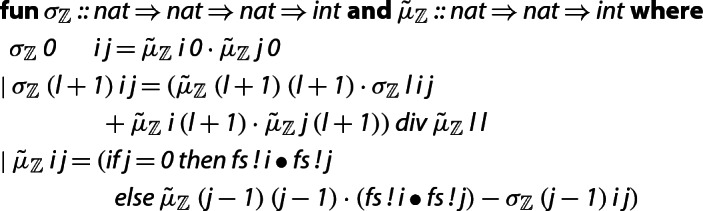
 Note that these functions only use integer arithmetic and therefore return a value of type . We then show that the new functions are equal to the ones defined previously. Here,  is a function that converts a number of type  into the corresponding number of type . For notational convenience, the indices of  are shifted by one with respect to the indices of . 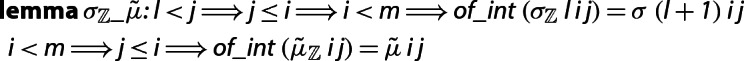


We then replace the repeated calls of $\tilde{\mu }_{\mathbb {Z}}$ by saving already computed values in an array for fast access. Furthermore, we rewrite $\sigma _\mathbb {Z}$ in a tail-recursive form, which completes the integer implementation of the algorithm for computing $\tilde{\mu }$.

Note that Algorithm 2 so far only computes the $\tilde{\mu }$-matrix. For completeness, we also formalize and verify an algorithm that computes the integer-valued multiples $\tilde{g_i} = d_ig_i$ of the GSO-vectors. Again, we first define the algorithm using rational numbers, then prove that all intermediate values are in fact integers, and finally refine the algorithm to an optimized and executable version that solely uses integer operations. A pseudo-code description is provided in the appendix as Algorithm 3.

### LLL Basis Reduction

We can now describe the formalization of an integer-only implementation of the LLL algorithm. For the version of the algorithm described in Sect. 3, we assumed that the GSO vectors and $\mu$-values are recomputed whenever the integer vectors *f* are changed. This made it easier to formalize the soundness proof, but as an implementation it would result in a severe computational overhead. Here we therefore assume that the algorithm keeps track of the required values and updates them whenever *f* is changed. This requires an extension of the soundness proof, since we now need to show that each change made to a value is consistent with what we would get if it were recomputed for the current value of *f*.

The version of the algorithm described in this section only stores *f*, the $\tilde{\mu }$-matrix, and the *d*-values, which, by lemma , are all integer values or integer vectors [25]. This integer representation will be the basis for our verified integer implementation of the LLL algorithm. To prove its soundness, we proceed similarly as for the GSO procedure: First we provide an implementation which still operates on rational numbers and uses field-division, then we use lemma  to implement and prove the soundness of an equivalent but efficient algorithm which only operates on integers.

The main additional difficulty in the soundness proof of the reduction algorithm is that we are now required to explicitly state and prove the effect of each computation that results in a value update. We illustrate this problem with lemma  (Sect. 4.2). The statement of this lemma only speaks about the effect, w.r.t. the invariant, of executing one while-loop iteration of Algorithm 1, but it does *not* provide results on how to update the $\tilde{\mu }$-values and the *d*-values. In order to prove such facts, we added several *computation* lemmas of the following form, which precisely specify how the values of interest are updated when performing a swap of $f_i$ and $f_{i-1}$, or when performing an update $f_i {:=} f_i - c \cdot f_j$. The newly computed values of  and  are marked with a  sign after the identifier. 
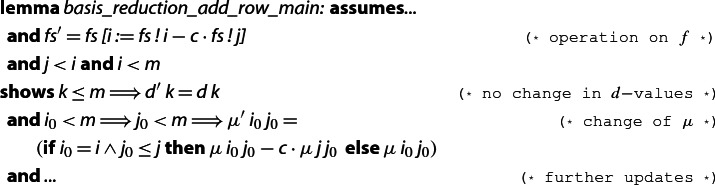


The computation lemma allows us to implement this part of the algorithm for various representations, i.e., the full lemma contains local updates for *f*, *g*, $\mu$, and *d*. Moreover, the lemma has actually been used to prove the soundness of the abstract algorithm: the precise description of the $\mu$-values allows us to easily establish the invariant in step 4 of Algorithm 1: if $c = \lfloor \mu _{i,j} \rceil$, then the new $\mu _{i,j}$-value will be small afterwards and only the $\mu _{i,j_0}$-entries with $j_0 \le j$ can change.

Whereas the computation lemmas such as the one above mainly speak about rational numbers and vectors, we further derive similar computation lemmas for the integer values $\tilde{\mu }$ and *d*, in such a way that the new values can be calculated based solely on the previous integer values of *f*, $\tilde{\mu }$, and *d*. At this point, we also replace field divisions by integer divisions; the corresponding soundness proofs heavily rely upon Lemma . As an example, the computation lemma for the swap operation of $f_{k-1}$ and $f_k$ provides the following equality for *d*, and a more complex one for the update of $\tilde{\mu }$.^4^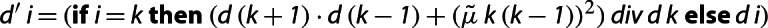 After having proved all the updates for $\tilde{\mu }$ and *d* when changing *f*, we implemented all the other expressions in Algorithm 1, e.g., $\lfloor \mu _{i,j} \rceil$, based on these integer values.

Finally, we plug everything together to obtain an efficient executable LLL algorithm—-—-that uses solely integer operations. It has the same structure as Algorithm 1 and therefore we are able to prove that the integer algorithm is a valid implementation of Algorithm 1, only the internal computations being different. The following lemma resides in the locale , but  takes the locale parameters $\alpha$ and  as explicit arguments, since we define it outside the locale as required by Isabelle’s code-generator [14]. 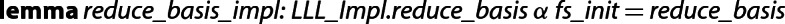


We also explain here the optimization of the algorithm, that was mentioned in Sect. 3.2: Whenever the variable *i* is decreased in one iteration of the main loop, the next loop iteration does not invoke lines 3–4 of Algorithm 1. Recall that these lines have the purpose of obtaining small $\mu _{i,j}$-values. However, when decreasing *i*, the $\mu _{i,j}$-values are already small. This can be deduced from the invariant of the previous iteration in combination with the computation lemmas for a swap.

In the appendix, Algorithm 5 shows a pseudo-code of .

## Complexity of the LLL Basis Reduction Algorithm

In this section we describe the formal proof of the polynomial-time complexity of our verified LLL implementation. This proof consists of two parts: showing that the number of arithmetic operations performed during the execution of the algorithm is bounded by a polynomial in the size of the input, and showing that the numbers on which the algorithm operates throughout its execution have a polynomially-bounded (bit-)size. These statements together give the desired complexity bound.

### Bounds on the Numbers in the LLL Algorithm

The computational cost of each of the basic arithmetic operations on integers ($+$, −, $\times$, $\div$) is obviously upper-bounded by a polynomial in the size of the largest operand. We are therefore interested in bounding the sizes of the various intermediate values that are computed throughout the execution of the algorithm. This is not a trivial task as already apparent in Examples 1 and 2, where we see that even the initial GSO computation can produce large numbers.

Our task is to formally derive bounds on $f_i$, ${\tilde{\mu }}_{i,j}$, $d_k$ and $g_i$, as well as on the auxiliary values computed by Algorithm 2. Although the implementation of Algorithm 2 computes neither $g_i$ nor ${\tilde{g}}_i$ throughout its execution, the proof of an upper bound on $\tilde{\mu }_{i,j}$ uses an upper bound on $g_i$.

Whereas the bounds for $g_i$ will be valid throughout the whole execution of the algorithm, the bounds for the $f_i$ depend on whether we are inside or outside the for-loop in lines 3–4 of Algorithm 1.

To formally verify bounds on the above values, we first define a stronger LLL-invariant which includes the conditions  and  and prove that it is indeed satisfied throughout the execution of the algorithm. Here, we define *N* as the maximum squared norm of the initial *f*-vectors. 
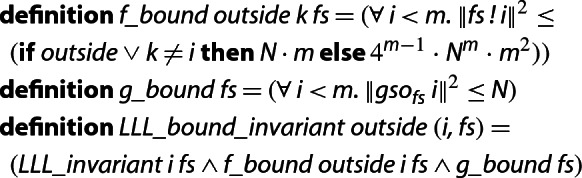


Note that  does not enforce a bound on the ${\tilde{\mu }}_{i,j}$, since such a bound can be derived from the bounds on *f*, *g*, and the Gramian determinants.

Based on the invariant, we first formally prove the bound $|\mu _{i,j}|^2 \le d_j \cdot |\!|f_i|\!|^2$ by closely following the proof from [29, Chapter 16]. It uses Cauchy’s inequality, which is a part of our vector library. The bound $d_k \le N^k$ on the Gramian determinant can be directly derived from the Lemma  and .

The previous two bounds clearly give an upper-bound on $\tilde{\mu }_{i,j} = d_{j+1}\mu _{i,j}$ in terms of *N*. Bounds on the intermediate values of $\sigma$ in Algorithm 2 are obtained via lemma  in Sect. 5.1. Finally, we show that all integer values *x* during the computing stay polynomial in *n*, *m*, and *M*, where *M* is the maximal absolute value within the initial *f*-vectors, satisfying $N \le M^2 \cdot n$. 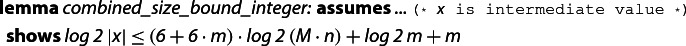


### A Formally Verified Bound on the Number of Arithmetic Operations

In this subsection we give an overview of the formal proof that our LLL implementation not only terminates on valid inputs, but does so after executing a number of arithmetic operations that is bounded by a polynomial in the size of the input.

The first step towards reasoning about the complexity is to extend the algorithm by annotating and collecting costs. In our cost model, we only count the number of arithmetic operations. To integrate this model formally, we use a lightweight approach that is similar to [11, 22]. It has the advantage of being easy to integrate on top of our formalization obtained so far, hence we did not try to incorporate alternative ways to track costs, e.g., via type systems [19].

- We use a type  to represent a result of type  in combination with a cost for computing the result.
- For every Isabelle function  that is used to define the LLL algorithm, we define a corresponding extended function . These extended functions use pattern matching to access the costs of sub-algorithms, and then return a pair where all costs are summed up.
- In order to state correctness, we define two selectors  and . Then soundness of  is split into two properties. The first one states that the result is correct: , and the second one provides a cost bound . We usually prove both statements within one inductive proof, where the reasoning for correct results is usually automatic.

We illustrate our approach using an example:  corresponds to lines 3–7 of Algorithm 2. 
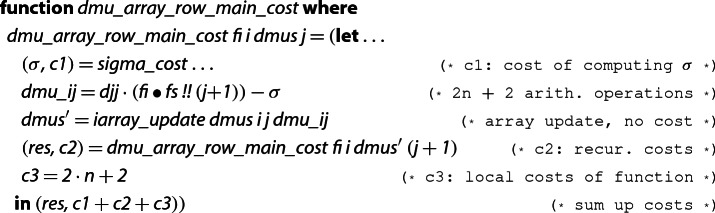


The function  is a typical example of cost-annotated function and works as follows: One part invokes sub-algorithms or makes a recursive call and extracts the cost by pattern matching on pairs ( and ), the other part does some local operations and manually annotates the costs for them (). Finally, the pair of the computed result and the total cost is returned. For all cost functions we prove that  is the value returned by the corresponding function.

To formally prove an upper bound on the cumulative cost of a run of the entire algorithm, we use the fact that  was defined as the logarithm of a product of Gramian determinants, together with the bound $d_k \le N^k \le (Mn)^{2k} \le (Mn)^{2m}$ from the previous subsection (where *M* was the maximum absolute value in the input vectors). This easily gives the desired polynomial bound: 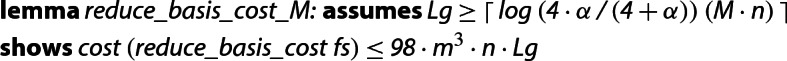


## Certifying Reduced Bases

In the previous sections we have seen a *verified* algorithm for computing a reduced basis of an arbitrary input lattice. The results of this development are twofold: first, one obtains a verified executable implementation of a lattice reduction algorithm; second, one can formally verify properties about lattice reductions, e.g., that a reduced basis always exists, that it can be computed in polynomial time, etc.. If one is only interested in the former property, namely having an implementation which never produces wrong results, there is also the alternative approach of certification.

The general idea behind certification is to combine a fast (but unverified) external algorithm $EA$, with a verified checker $VC$. The workflow is as follows. One invokes algorithm $EA$ in order to obtain a result in combination with a certificate. This certificate must contain enough auxiliary information so that $VC$ can check whether the result is indeed a correct result for the given input.

In this section we will now instantiate the general certification idea for the case of lattice reduction. The input is as before, i.e., a linearly independent list of basis vectors (represented as a matrix *F* whose rows are the vectors) and an approximation factor $\alpha$. For the fast algorithm we can in principle use any external tool for lattice reduction. However, just computing a reduced basis *R* does not suffice. For instance, it is not enough to return the reduced basis of Example 3 for Example 1, since one needs to ensure that both bases span the same lattice. Hence, we need a certificate that allows us to efficiently check that the lattice of the input *I* is identical to that of the result *R*. To that end, we require that the external tool provides as certificate *C* two integer matrices *U* and *V* such that

4 $$\begin{aligned} F = U \times R \text { and } R = V \times F, \end{aligned}$$

and indeed, current LLL implementations can already provide these certificates.

Obviously, condition (4) can be efficiently checked, given the four matrices *F*, *R*, *U*, and *V*. Moreover, we formally prove that whenever (4) is valid, *F* and *R* span the same lattice, and furthermore, whenever *F* represents a list of linearly independent vectors, so does *R*. It remains to have a certifier to check whether *R* is indeed reduced w.r.t. $\alpha$, cf. Definition 1. In principle, this can be done easily and efficiently via Algorithm 2: the algorithm computes in particular all $d_i$-values, from which one can immediately compute the norms of the GSO. However, our actual certifier just invokes the full verified lattice reduction algorithm on *R* and $\alpha$ to obtain the final result. This makes the connection between the certifier and the external algorithm less brittle and in particular, allows the use of different approximation factors. If $EA$ internally^5^ uses a better approximation factor than $\alpha$, then in the LLL invocation during certification, only the GSO will be computed, and then it is checked that all $\mu$-values are small and that the norms of $g_i$ are nearly sorted. In this case, no swaps in line 6 of Algorithm 1 will occur. If $EA$ uses a smaller approximation factor than $\alpha$, then $EA$ simply does more work than required, certification is unaffected. More importantly, the case where $EA$ uses a larger approximation factor than $\alpha$ is also permitted: in this case, the basis returned by $EA$ will be further reduced w.r.t. $\alpha$ as needed by the verified algorithm.

The actual implementation in Isabelle looks as follows.^6^ Here,  is an unspecified Isabelle constant, which can be implemented arbitrarily in the generated code; only the type is fixed.  is a constant that is translated into an error message in the generated code and ignores its second argument. 
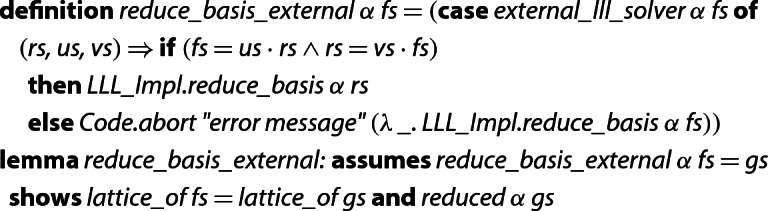


Note that the else-branch of  is logically equivalent to . This is the reason why the soundness lemma for  can be proven, even when the external solver produces a wrong result.

Overall, the certification approach for basis reduction looks quite attractive. As we will see in the next section, it is faster than the fully verified implementation, and has the same soundness property, cf. lemma  in Sect. 4.2. Still,  should be used with great care, since one important aspect is typically lost when using an external tool for basis reduction: the Isabelle function  does not necessarily behave like a mathematical function anymore: invoking the function twice on the same input might deliver different results, if the external tool is randomized or parallelized.

## Experiments on LLL Basis Reduction

We formalized the LLL lattice reduction algorithm in a way that allows us to use Isabelle’s code generator [14] and, hence, to compare our verified implementation to other implementations in terms of efficiency. We tested five different configurations.

- **verified**: In this configuration we run our fully verified implementation of the LLL algorithm. Here, we fix $\alpha = \frac{3}{2}$, we map Isabelle’s integer operations onto the unbounded integer operations of the target language Haskell, and we compile the code with ghc version 8.2.1 using the -O2 parameter.
- **Mathematica**: In this configuration we invoke the LatticeReduce procedure of Mathematica version 11.3 [30]. The documentation does not specify the value of $\alpha$, but mentions that Storjohann’s variant [25] of the LLL basis reduction algorithm is implemented. The (polynomial) complexity of this variant is one degree lower than that of our algorithm.
- **fplll**: Here we are using fplll version 5.2.1 to reduce lattices. It implements floating-point variants of the LLL algorithm, and we run it with $\alpha = \frac{3}{2}$.
- **fplll+certificate**: This is the same as **fplll**, except that fplll is configured in such a way that a certificate according to Sect. 7 will be computed (the matrices *U* and *V* form the certificate that is returned together with *R*).
- **certified**: This configuration is the certification approach of Sect. 7. We invoke  in the same way as in the **verified** configuration, where **fplll+certificate** is used as an external tool.

We tested all configurations on example lattices arising from random polynomial factorization problems. Here, the parameter *n* specifies the size of the input lattices in three ways: it is the number of input vectors, the dimension of each input vector, and the number of digits of the coefficients of the input vectors. Hence, the input size is cubic in *n*.

**Fig. 1:**
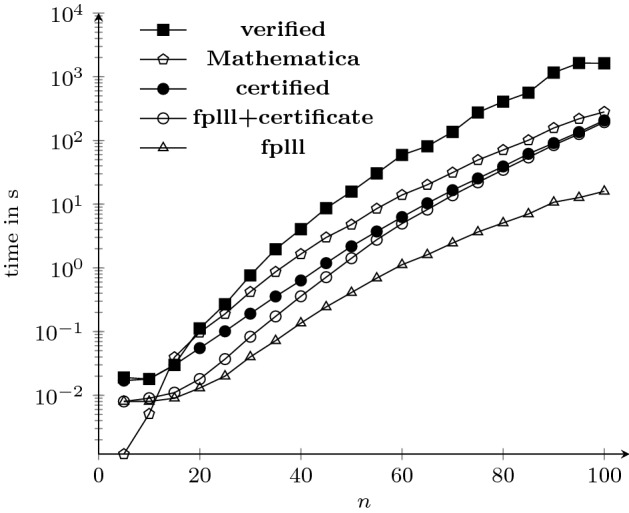
Efficiency of LLL implementations on lattices from polynomial factorization

We tested values of *n* between 5 and 100. All experiments were run on an iMacPro with a 3.2 GHz Intel Xeon W running macOS 10.14.3 and the results are illustrated in Fig. 1 and Table 1. In Fig. 1, all verified results are indicated by solid marks, and all configurations where the results are not verified are indicated with blank marks. Both the generated code and our experimental data are available at the following website: https://doi.org/10.5281/zenodo.2636366.

**Table 1:** Execution time of LLL implementations

Configuration	Total time (in s)
**verified**	6006.4
**Mathematica**	962.0
**certified**	600.4
**fplll+certificate**	547.6
**fplll**	61.9

Although the **verified** configuration is the slowest one, it takes 6 006 seconds in total on these examples, which is a big improvement over the previous verified implementation [8], which requires 2.6 million seconds in total. Moreover, the **certified** configuration based on fplll is even faster than **Mathematica**, and additionally provides results that are formally verified to be correct.

It is interesting to observe the overhead of certification. One can see that checking the certificate is really fast, since there is only 10 % difference in the runtime between **fplll+certificate** and **certified**. Here, the fast implementation of the GSO algorithm is essential. However, producing the certificate induces quite some overhead, cf. the difference between **fplll+certificate** and **fplll**. Finally, the experiments also clearly illustrate that our verified algorithm cannot compete against floating-point implementations of the LLL algorithm.

To summarize, in addition to having the advantage of delivering provably correct results, both our verified and our certified implementation are usable in practice, in contrast to our previous verified implementation. Besides efficiency, it is worth mentioning that we did not find bugs in fplll’s or Mathematica’s implementation: each certificate of **fplll+certificate** has been accepted and the short vectors that are generated by fplll have always been as short as our verified ones. Moreover, the norms of the short vectors produced by Mathematica are similar to our verified ones, differing by a factor of at most 2.

## Polynomial Factorization via Short Vectors

In this section we formalize one of the important applications of the LLL algorithm: polynomial-time algorithms for polynomial factorization. In Sect. 9.1 we first describe the key idea on how the LLL algorithm helps to factor integer polynomials, following the textbook [29, Chapters 16.4–16.5]. Section 9.2 presents the formalization of some necessary results. In combination with our previous work [7], this is sufficient for obtaining a polynomial-time algorithm to factor arbitrary integer polynomials, whose formalization is presented in Section 9.3. When attempting to directly verify the factorization algorithm in the above-mentioned textbook (Algorithm 16.22 in [29]), it turned out that the original algorithm has a flaw that made the algorithm return incorrect results on certain inputs. The details and a corrected version are provided in Sect. 9.4.

### Short Vectors for Polynomial Factorization

The common structure of a modern factorization algorithm for square-free primitive polynomials in $\mathbb {Z}[x]$ is as follows:

1. A prime *p* and exponent *l* are chosen depending on the input polynomial *f*.
2. A factorization of *f* over $\mathbb {Z}_p[x]$ is computed.
3. Hensel lifting is performed to lift the factorization to $\mathbb {Z}_{p^l}[x]$.
4. The factorization $f=\prod _i f_i \in \mathbb {Z}[x]$ is reconstructed where each $f_i$ corresponds to the product of one or more factors of *f* in $\mathbb {Z}_{p^l}[x]$.

In a previous work [7], we formalized the Berlekamp–Zassenhaus algorithm, which follows the structure presented above, where step 4 runs in exponential time. The use of the LLL algorithm allows us to derive a polynomial-time algorithm for the reconstruction phase.^7^ In order to reconstruct the factors in $\mathbb {Z}[x]$ of a polynomial *f*, by steps 1–3 we compute a *modular* factorization of *f* into several monic factors $u_i$:  modulo *m* where $m = p^l$ is some prime power given in step 1.

The intuitive idea underlying why lattices and short vectors can be used to factor polynomials follows. We want to determine a non-trivial factor *h* of *f* which shares a common modular factor *u*, i.e., both *h* and *f* are divided by *u* modulo $p^l$. This means that *h* belongs to a certain lattice. The condition that *h* is a factor of *f* means that the coefficients of *h* are relatively small. So, we must look for a *small* element (a short vector) in that lattice, which can be done by means of the LLL algorithm. This allows us to determine *h*.

More concretely, the key is the following lemma.

#### Lemma 1

([29, Lemma 16.20]) Let *f*, *g*, *u* be non-constant integer polynomials. Let *u* be monic. If *u* divides *f* modulo *m*, *u* divides *g* modulo *m*, and , then  is non-constant.

Let *f* be a polynomial of degree *n*. Let *u* be any degree-*d* factor of *f* modulo *m*. Now assume that *f* is reducible, so that $f = f_1 \cdot f_2$, where w.l.o.g. we may assume that *u* divides $f_1$ modulo *m* and that . Let $L_{u,k}$ be the lattice of all polynomials of degree below $d+k$ which are divisible by *u* modulo *m*. As , clearly $f_1 \in L_{u, n - d}$.

In order to instantiate Lemma 1, it now suffices to take *g* as the polynomial corresponding to any short vector in $L_{u,n-d}$: *u* divides *g* modulo *m* by definition of $L_{u,n-d}$ and moreover . The short vector requirement provides an upper bound to satisfy the assumption .

5 $$\begin{aligned}&|\!|g|\!| \le 2^{(n - 1)/2} \cdot |\!|f_1|\!| \le 2^{(n - 1)/2} \cdot 2^{n-1} |\!|f|\!| = 2^{3(n-1)/2} |\!|f|\!| \end{aligned}$$

6The first inequality in (5) is the short vector approximation ($f_1 \in L_{u,n-d}$). The second inequality in (5) is Mignotte’s factor bound ($f_1$ is a factor of *f*). Mignotte’s factor bound and (5) are used in (6) as approximations of $|\!|f_1|\!|$ and $|\!|g|\!|$, respectively. Hence, if *l* is chosen such that $m = p^l > |\!|f|\!|^{2(n-1)} \cdot 2^{5(n - 1)^2/2}$, then all preconditions of Lemma 1 are satisfied, and  is a non-constant factor of *f*. Since $f_1$ divides *f*, also  is a non-constant factor of *f*. Moreover, the degree of *h* is strictly less than *n*, and so *h* is a proper factor of *f*.

### Formalization of the Key Results

Here we present the formalization of two items that are essential for relating lattices and factors of polynomials: Lemma 1 and the lattice $L_{u,k}$.

To prove Lemma 1, we partially follow the textbook, although we do the final reasoning by means of some properties of resultants which were already proved in the previous development of algebraic numbers [16]. We also formalize Hadamard’s inequality, which states that for any square matrix *A* having rows $v_i$ we have . Essentially, the proof of Lemma 1 consists of showing that the resultant of *f* and *g* is 0, and then deduce . We omit the detailed proof; a formalized version can be found in the sources.

To define the lattice $L_{u,k}$ for a degree-*d* polynomial *u* and integer *k*, we give a basis $v_0,\dots ,v_{k+d-1}$ of the lattice $L_{u,k}$ such that each $v_i$ is the $(k+d)$-dimensional vector corresponding to polynomial $u(x) \cdot x^i$ if $i<k$, and to the monomial $m\cdot x^{k+d-i}$ if $k \le i < k+d$.

We define the basis in Isabelle/HOL as  as follows: 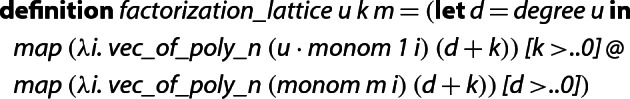 Here,  denotes the list of natural numbers descending from $a-1$ to *b* (with $a>b$),  denotes the monomial $ax^b$, and  is a function that transforms a polynomial *p* into a vector of dimension *n* with coefficients in the reverse order and completing with zeroes if necessary. We use it to identify an integer polynomial *f* of degree $< n$ with its coefficient vector in ${\mathbb {Z}}^{n}$. We also define its inverse operation, which transforms a vector into a polynomial, as .

To visualize the definition, for $u(x) = \sum _{i=0}^d u_i x^i$ we have7and  is precisely the basis $(f_0,f_1,f_2)$ of Example 1.

There are some important facts that we must prove about .

*ukm* is a list of linearly independent vectors, as required for applying the LLL algorithm in order to find a short vector in $L_{u,k}$.
- $L_{u,k}$ characterizes the polynomials which have *u* as a factor modulo *m*:  That is, any polynomial that satisfies the right-hand side can be transformed into a vector that can be expressed as an integer linear combination of the vectors of . Similarly, any vector in the lattice $L_{u,k}$ can be expressed as an integer linear combination of  and corresponds to a polynomial of degree less than $k+d$ that is divisible by *u* modulo *m*.

The first property is a consequence of the obvious fact that the matrix *S* in (7) is upper triangular, and that its diagonal entries are non-zero if both *u* and *m* are non-zero. Thus, the vectors in *ukm* are linearly independent.

Next, we look at the second property. For one direction, we see the matrix *S* as (a generalization of) the *Sylvester matrix* of the polynomial *u* and constant polynomial *m*. Then we generalize an existing formalization about Sylvester matrices as follows: 
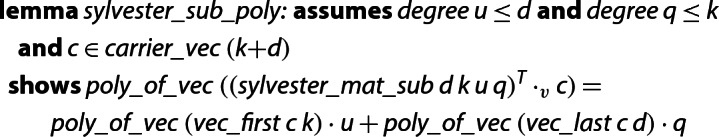
 We instantiate  by the constant polynomial *m*. So for every $c \in \mathbb {Z}^{k+d}$ we getfor some polynomials *r* and *s*. As every $g \in L_{u,k}$ is represented as $S^{{\mathsf {T}}} c$ for some integer coefficient vector $c \in \mathbb {Z}^{k+d}$, we conclude that every $g \in L_{u,k}$ is divisible by *u* modulo *m*. The other direction requires the use of division with remainder by the monic polynomial *u*. Although we closely follow the textbook, the actual formalization of these reasonings requires some more tedious work, namely the connection between the matrix-times-vector multiplication of Matrix.thy (denoted by  in the formalization) and linear combinations () of HOL-Algebra.

### A Verified Factorization Algorithm

Once the key results, namely Lemma 1 and properties about the lattice $L_{u,k}$, are proved, we implement an algorithm for the reconstruction of factors within a context that fixes  and . The simplified definition looks as follows. 
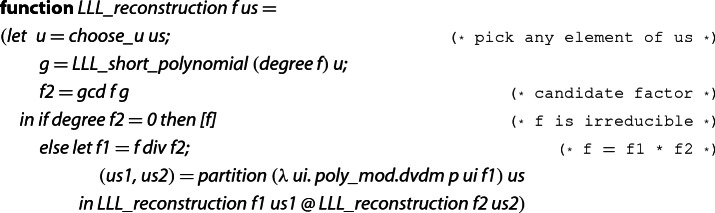


 is a recursive function which receives two parameters: the polynomial  that has to be factored, and the list  of modular factors of the polynomial .  computes a short vector (and transforms it into a polynomial) in the lattice generated by a basis for $L_{u,k}$ and suitable *k*, that is, . We collect the elements of  that divide  modulo  into the list , and the rest into .  returns the list of irreducible factors of . Termination follows from the fact that the degree decreases, that is, in each step the degree of both  and  is strictly less than the degree of .

In order to formally verify the correctness of the reconstruction algorithm for a polynomial  we use the following invariants for each invocation of , where  is an intermediate non-constant factor of . Here some properties are formulated solely via , so they are trivially invariant, and then corresponding properties are derived locally for  by using that  is a factor of .

divides
is the unique modular factorization of  modulo
and  are coprime, and  is square-free in
is sufficiently large:  where

Concerning complexity, it is easy to see that if a polynomial splits into *i* factors, then  invokes the short vector computation $i + (i-1)$ times: $i-1$ invocations are used to split the polynomial into the *i* irreducible factors, and for each of these factors one invocation is required to finally detect irreducibility.

Finally, we combine the new reconstruction algorithm with existing results presented in the Berlekamp–Zassenhaus development to get a polynomial-time factorization algorithm for square-free and primitive polynomials. 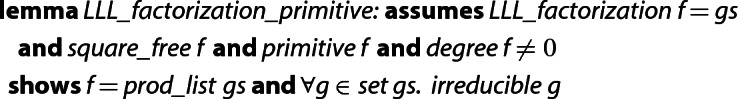


We further combine this algorithm with a pre-processing algorithm also from our earlier work [7]. This pre-processing splits a polynomial *f* into $c \cdot f_1^1 \cdot \ldots \cdot f_k^k$ where *c* is the content of *f* which is not further factored (see Sect. 2). Each $f_i$ is primitive and square-free, and will then be passed to . The combined algorithm factors arbitrary univariate integer polynomials into its content and a list of irreducible polynomials.

The Berlekamp–Zassenhaus algorithm has worst-case exponential complexity, e.g., exhibited on Swinnerton–Dyer polynomials. Still it is a practical algorithm, since it has polynomial average complexity [5], and this average complexity is smaller than the complexity of the LLL-based algorithm, cf. [29, Ch. 15 and 16]. Therefore, it is no surprise that our verified Berlekamp–Zassenhaus algorithm [7] significantly outperforms the verified LLL-based factorization algorithm on random polynomials, as it factors, within one minute, polynomials that the LLL-based algorithm fails to factor within any reasonable amount of time.

### The Factorization Algorithm in the Textbook Modern Computer Algebra

In the previous section we have chosen the lattice $L_{u,k}$ for $k = n-d$, in order to find a polynomial *h* that is a proper factor of *f*. This has the disadvantage that *h* is not necessarily irreducible. By contrast, Algorithm 16.22 from the textbook tries to directly find *irreducible* factors by iteratively searching for factors w.r.t. the lattices $L_{u,k}$ for increasing *k* from 1 up to $n - d$.
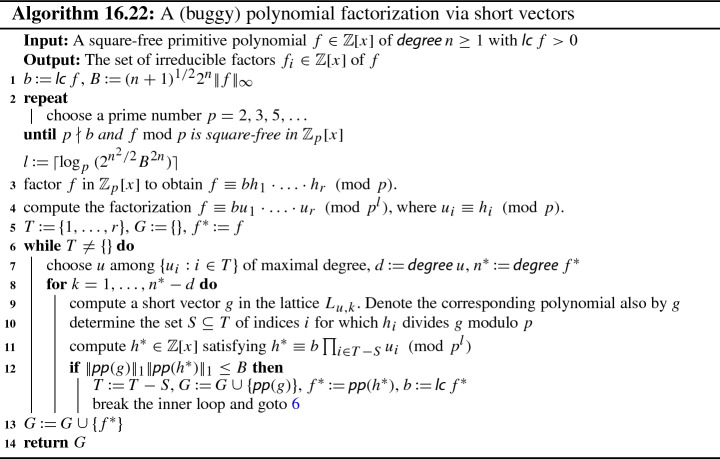


The max-norm of a polynomial $f(x) = \sum _{i=0}^n c_i x^i$ is defined to be $|\!|f|\!|_\infty = \max \{|c_0|,\dots ,|c_n|\}$, the 1-norm is $|\!|f|\!|_1=\sum _{i=0}^{n} |c_i|$ and  is the primitive part of *f*, i.e., the quotient of the polynomial *f* by its content.

Let us note that Algorithm 16.22 also follows the common structure of a modern factorization algorithm; indeed, the reconstruction phase corresponds to steps 5-13. Once again, the idea behind this reconstruction phase is to find irreducible factors via Lemma 1 and short vectors in the lattice $L_{u,k}$. However, this part of the algorithm (concretely, the inner loop presented at step 8) can return erroneous calculations, and some modifications are required to make it sound.

The textbook proposes the following invariants to the reconstruction phase:

- $$f^{{\text {*}}} \equiv b \prod _{i\in T} u_i \pmod {p^l}$$,
,
- $$f=\pm f^{{\text {*}}} \prod _{g \in G} g$$, and
- each polynomial in *G* is irreducible.

While the arguments given in the textbook and the provided invariants all look reasonable, the attempt to formalize them in Isabelle runs into obstacles when one tries to prove that the content of the polynomial *g* in step 9 is not divisible by the chosen prime *p*. In fact, this is not necessarily true.

The first problem occurs if the content of *g* is divisible by *p*. Consider $f_1 = x^{12}+x^{10}+x^8+x^5+x^4+1$ and $f_2 = x$. When trying to factor $f = f_1 \cdot f_2$, then $p = 2$ is chosen, and in step 9 the short vector computation is invoked for a modular factor *u* of degree 9 where $L_{u,4}$ contains $f_1$. Since $f_1$ itself is a shortest vector, $g = p \cdot f_1$ is a short vector: the approximation quality permits any vector of $L_{u,4}$ of norm at most . For this valid choice of *g*, the result of Algorithm 16.22 will be the non-factorization $f = f_1 \cdot 1$.

The authors of the textbook agreed that this problem can occur. The flaw itself is easily fixed by modifying step 10 toA potential second problem revealed by our formalization work, is that if *g* is divisible not only by *p* but also by $p^l$, Algorithm 16.22 will still return a wrong result (even with step 10 modified). Therefore, we modify the condition in step 12 of the factorization algorithm and additionally demand , and then prove that the resulting algorithm is sound. Unlike the first problem, we did not establish whether or not this second problem can actually occur.

Regarding to the implementation, apart from the required modifications to make Algorithm 16.22 sound, we also integrate some changes and optimizations:

- We improve the bound *B* at step 1 with respect to the one used in the textbook.
- We test a necessary criterion whether a factor of degree $d+k$ is possible, before performing any short vector computations in step 9. This is done by computing all possible degrees of products of the modular factors $\prod _{i \in I}u_i$.
- We dynamically adjust the modulus to compute short vectors in smaller lattices: Directly before step 9 we compute a new bound $B'$ and a new exponent $l'$ depending on the current polynomial $f^{\text {*}}$ and the degree $d+k$, instead of using the ones computed in steps 1-2, which depend on the input polynomial *f* and its degree *n*. This means that the new exponent $l'$ can be smaller than *l* (otherwise, we follow the computations with *l*), and the short vector computation of step 9 will perform operations in a lattice with smaller values.
- We check divisibility instead of norm-inequality in step 12. To be more precise, we test  instead of the condition in step 12. If this new condition holds, then $h^{\text {*}}$ is not computed as in step 11, but directly as the result of dividing *f* by .

The interested reader can explore the implementation and the soundness proof of the modified algorithm in the file Factorization_Algorithm_16_22.thy of our AFP entry [9]. The file Modern_Computer_Algebra_Problem.thy in the same entry shows some examples of erroneous outputs of the textbook algorithm. A pseudo-code version of the fixed algorithm is detailed in the appendix as Algorithm 4.

## Conclusion

We formalized an efficient version of the LLL algorithm for finding a basis consisting of short, nearly orthogonal vectors of an integer lattice in Isabelle/HOL. In addition, we provided a formal proof of its polynomial-time complexity. Our verified algorithm shows a remarkable performance. In order to improve the performance even further, we also provided a certified approach: we developed a verified checker that uses a fast untrusted lattice reduction algorithm based on floating-point arithmetic. This approach is also formally proven correct, and runs even faster than Mathematica.

One of the most famous application of the LLL algorithm has also been formalized, namely a factorization algorithm for integer polynomials which runs in polynomial time. The work is based on our previous formalization of the Berlekamp–Zassenhaus factorization algorithm, where the exponential reconstruction phase is replaced by the polynomial-time lattice-reduction algorithm.

The whole formalization consists of 14 811 lines of code, it took about 23 person months to formalize approximately 24 pages of textbooks and research articles. The de Bruijn factor is about 17, mainly due to the informal proofs presented in the textbooks. The set-based matrix- and vector-library has been essential for dealing with matrices of varying sizes, but is cumbersome to use, because the proof automation in the set-based setting in Isabelle/HOL is not as developed as for the type-based setting, and its usage requires additional statements such as vectors being of the right dimension. During the development we also extended six different AFP entries, e.g., we added Laplace’s expansion rule and Cramer’s rule for determinants over arbitrary rings to the vector- and matrix-library.

As far as we know, this is the first formalization of the LLL algorithm and its application to factor polynomials in any theorem prover. This formalization led us to find and correct a major flaw in a textbook.

One way to further build on this work would be to formalize a fast polynomial factorization algorithm that uses the LLL basis reduction algorithm as a subroutine, such as van Hoeij’s algorithm [28], which would make full use of the efficiency of our current implementation.

## Algorithms

In the following verified algorithm for computing the GSO,  is vector-by-scalar division on integers. We proved that each invocation of the division is exact.

Algorithm 4 presents the fixed version of Algorithm 16.22, including the improvements described in Sect. 9.4.

Algorithm 5 shows the verified integer implementation of the LLL algorithm. The line numbers are chosen in such a way that they correspond to the line numbers in the LLL implementation provided in Algorithm 1. Most of the remaining code is executed in order to keep the values of *d* and $\tilde{\mu }$ up-to-date. Our functional implementation of the algorithm differs in one aspect from the pseudo-code, namely the update of $\tilde{\mu }$ between lines 5 and 6 is done by constructing a completely new $\tilde{\mu }$-matrix in our code. The problem is that we are restricted to immutable data structures and cannot update the $\tilde{\mu }$-matrix in place. Hence, our implementation of the swap-step requires quadratically many operations, whereas an implementation with mutable arrays only needs linearly many operations for a swap.

## Footnotes

1 Choosing $\alpha = \frac{4}{3}$ is also permitted, but then the polynomial running time is not guaranteed.

2 $\frac{4\alpha }{4 + \alpha } = 1$ for $\alpha = \frac{4}{3}$ and in that case one has to drop the logarithm from the measure.

3 Cramer’s lemma (also known as Cramer’s rule) states that, given a system of linear equations $Ax=b$, the solution can be computed via the equality ${{\text {det}} A} \cdot x_j = {\text {det}} A_j$, where $A_j$ is the matrix obtained from *A* by replacing the *j*-th column with the vector *b*.

4 The updates for $\tilde{\mu }_{i,j}$ consider 5 different cases depending on the relations between *i*, *j*, *k*.

5 Whether one can specify the approximation factor at all, depends on the interface of the external lattice reduction algorithm.

6 For the sake of readability, we omit some necessary conversions between lists and vectors as well as some checks on matrix-dimensions.

7 We did not formally prove the complexity bound for either of the factorization algorithms.
